# Modeling of the Influence of Chemical Composition, Sintering Temperature, Density, and Thickness in the Light Transmittance of Four Zirconia Dental Prostheses

**DOI:** 10.3390/ma12162529

**Published:** 2019-08-08

**Authors:** Yuri Resende Fonseca, Carlos Nelson Elias, Sergio Neves Monteiro, Heraldo Elias Salomão dos Santos, Claudinei dos Santos

**Affiliations:** 1 Instituto Militar de Engenharia, Materials Science Department, Praça General Tibúrcio, 80, Praia Vermelha, Rio de Janeiro, RJ 22290-270, Brazil; 2 Faculdade de Tecnologia, Universidade Estadual do Rio de Janeiro, Rod. Presidente Dutra, km 298, Polo Industrial, Resende, RJ 27537-000, Brazil

**Keywords:** zirconia, transmittance, translucency, prosthetic dentistry

## Abstract

Research has increasingly been conducted to improve the toughness and aesthetics of zirconium oxide (zirconia) used in prosthetic dentistry. However, the balance between better mechanical properties and greater translucency, to ensure aesthetic requirements, is still a challenge in the development of a novel zirconia for dentistry applications. This study evaluated the transmittance of visible light for four types of pre-sintered zirconia blocks used in dentistry (3Y-SBE, Zpex, Zpex-4, and Zpex-Smile). The objective is to analyze the simultaneous influence of sintering temperature, in the range of 1450–1560 °C, as well as the chemical composition (%Y_2_O_3_), density, and thickness (1.0, 1.3, 1.6, and 2.0 mm) in the zirconia’s transmittance. To evaluate the interactive influence of these variables, a statistical learning model based on gradient boosting is applied. The results showed that the effect of the sintering temperature has an optimal (maximum transmittance) point. Increasing the temperature beyond this point reduces the transmittance of the material for all types of zirconia. Moreover, the optimal transmittance point is affected by the chemical composition of each type of zirconia. In addition, the results showed that the transmittance of all types of zirconia had an inverse relationship with the density, zirconia Zpex-Smile being the most sensitive to this parameter. Furthermore, the transmittance of 3Y-SBE, Zpex, and Zpex-4 decreases approximately linearly with the specimen thickness, whereas zirconia Zpex-Smile has a sublinear decrease, which is expected due to the optical isotropy of the cubic phase.

## 1. Introduction

Pure zirconia, (ZrO_2_, ceramic) has three polymorphic phases (monoclinic, tetragonal, and cubic) stable in increasing order of temperature. By adding a certain percentage of dopant oxide, such as cerium oxide (CeO_2_), magnesium oxide (MgO), and most commonly yttrium oxide or yttria (Y_2_O_3_), it is possible to induce metastability in the tetragonal phase at room temperature, obtaining the tetragonal zirconia [1]. The polycrystalline zirconia, most used as dental prosthesis, is that stabilized with Y_2_O_3_, known as nY-TZP, where n is the yttria percentage.

The Y-TZP has good toughness, which is associated with the transformation of the metastable tetragonal phase to monoclinic (*t→m*) induced by stress. This phase transformation is accompanied by a volumetric expansion by 5%, which generates compression stress at the crack tip and impairs its propagation, thereby increasing the material’s toughness [1,2,3].

Y-TZP is currently being considered for use in prosthetic dentistry owing to its favorable toughness [3,4,5]. However, for this application, other properties are also required, particularly translucency for aesthetic reasons [5,6]. Traditionally, it is expected that as grain size increases, the zirconia translucency increases, since this strategy decreases grain boundary density and reduces refraction [7,8]. However, if the scattering phenomenon occurs in a grain substantially smaller than the wavelength of incident light, an inverse relationship between transmittance and grain size can be observed. This phenomenon is modeled empirically using the Rayleigh equation and was perfectly adjusted for alumina [7].

Zhang [7] applied the Rayleigh model to 3Y-TZP zirconia to correlate the translucency, grain size, and thickness of the zirconia prosthesis. The results showed that the actual data differ slightly from the simulations, suggesting that, unlike alumina, the Rayleigh model may not be the most suitable for the analysis of nanostructured tetragonal zirconia.

The mechanical properties and translucency of zirconia are influenced by several parameters, including relative green body density, sintering temperature, and/or chemical composition. The sintering temperature influences the grain size and zirconia density. The density of grain boundaries alters the optical scattering, opacity, and mechanical performance of zirconia. The difficulty of the analysis involving the simultaneous influence of all parameters and linearity in behavior is not observed. There is also synergism among properties. Due to the complexity of the possible interactions of these parameters, it is difficult to predict the behavior of the zirconia with new chemical compositions or sintering temperatures. Consequently, the development of a novel zirconia combining desirable high mechanical properties and translucency is still a complex open challenge.

## 2. Materials and Methods

In the present work, pre-sintered zirconia blocks produced by ProtMat Materiais Avançados (Juiz de Fora, MG, Brazil) were used. Zirconia powders with commercial designations TZ-3YSBE, Zpex, Zpex-4, and Zpex-Smile, supplied by TOSOH-Japan (Tokyo, Japan), were used to produce the blocks. The powders were uniaxially pressed at 80 to 90 MPa, according to TOSOH instructions.

Table 1 shows the chemical compositions of the zirconia powders and respective pre-sintered block designations. As shown in this table, the composition of powders basically differs from Y_2_O_3_ content which is 3 mol % (5.2 wt %) for TZ-3YSBE and Zpex, 4 mol % (7.0 wt %) for Zpex 4, and 5.2 mol % (9.3 wt %) for Zpex-Smile.

To analyze the influence of the chemical composition (Y_2_O_3_ content), sintering temperature, density, wavelength, and thickness on the material transmittance, 160 specimens of each type of zirconia were prepared. The specimens were sintered at 1700 °C in the NBC model furnace, from Nobody Materials Science and Technology Co. (Zhengzhou, China). All specimens were sintered at a heating and cooling rate of 5 °C/min. Final plateau sintering temperatures were 1450, 1500, 1530, and 1560 °C. The sintering time was 120 min at plateau). After sintering, samples had thicknesses of 1.00, 1.25, 1.50, and 1.75 mm. The thickness measurements were verified with micrometer, and a maximum variation of 0.01 mm was observed.

The level of porosity directly affects the scattering of light in the zirconia. It is also inversely proportional to the relative density of every ceramic material [7]. Therefore, the bulk density was used to account for the influence of the density (consequently the porosity) on the zirconia transmittance. Moreover, to separate the effect of the sintering temperature from the density, different green densities were used to produce specimens of different chemical compositions and the same sintering temperature.

The specimens’ densities were quantified for the analysis of the influence on transmittance. The bulk density was determined by the Archimedes principle [9]. The masses of ten samples were taken for each group using the Gehaka model AG200 scale (Electro-Eletronica Gehaka Ltd.a., São Paulo, SP, Brazil) with an accuracy of 10^−4^ g. The mass was measured with the specimen fully immersed in a beaker containing distilled water and then measured with the sample resting on the bottom of the beaker.

The crystalline phases present in the sintered ceramics were performed by X-ray diffraction (XRD) using a Panalytical^®^ (Almelo, Netherlands) Empyrean diffractometer, with Cu-Kα radiation, 2θ range of 20–80°, a step width of 0.02°, and an exposure time of 5 s. The crystalline peaks were identified by comparison with the JCPDS data files using X’Pert HighScore Plus software and quantification was estimated according Scott, Matsui et al. and Chen et al. [10,11,12].

To obtain the percentage of transmittance, the experiment was performed on a spectrophotometer that operates in the wavelength range of visible light (CM-3700d, Konica Minolta, Tokyo, Japan). The light source of the equipment is a xenon arc lamp, and the generated data were computed in the wavelength range of 360–740 nm, at intervals of 10 nm. The transmittance test was performed using a standard two-blot black-and-white card (AG-5330, BYK Gardner, Columbia, MD, USA). The results obtained from the test correspond to transmittance values at every 10 nm, between 370 and 740 nm. According to the Technical Standard ISO 4007: 2018, there are no precise limits for the spectral range of visible radiation since they depend upon the amount of radiant power reaching the observer’s retina. The lower limit is generally taken between 360 and 400 nm and the upper limit between 760 and 830 nm. The limits of the visible spectrum are usually taken to be 380 nm (blue violet) to 780 nm (red).

For characterizing and quantifying the surface roughness, step heights, critical dimensions, and other topographical features, the NewView™ 7100 (Zygo Company, Middlefield, CT, USA) light interferometer (profilometer) was used. The NewView™ 7100 provides affordable versatility in non-contact optical 3D surface profiling [13]. The parameters for numerical roughness characterization were arithmetic mean of the absolute values of roughness (Ra), the largest valley depth value (PV), peak (Peak), and valley.

## 3. Statistical Modeling

The analysis of the influence of the various parameters and the zirconia processing in the transmittance is difficult due to the interaction among several variables. The attempt to employ parametric models that isolate one effect can generate a bias of omitted variables due to simplifications in the model. In addition, the nonlinear influence of each variable requires complex mathematical models to represent the joint dependence of all variables studied.

Recent parametric approaches, such as the use of Rayleigh scattering model, require strong assumptions with respect to the shape of grains, pores, and particles sizes. Alternatives, such as the use of the Mie scattering model, makes use of similar assumptions and involves simulations and numerical solutions to solve electromagnetic waves equations.

In the present work, we used a statistical learning model based on gradient boosting [14,15]. The technique has been widely used in fields such as computer science, economics, genetics, and others.

The model work as follows: we assume that the transmittance is described by a set of measured variables that affects directly or indirectly the transmittance of zirconia. In this case, the variables are density, sintering temperature, composition, and thickness. It is important that these variables also define other variables that were not measured, such as grain size. Therefore, since these variables are all defined implicitly, the model will be able to capture the interaction among them and the effect in the transmittance of zirconia.

To estimate the function, we assume that it can be approximated by a combination of simpler functions that are easier to estimate, called base learners [14]. The method works iteratively. At each step of the approximation, a new base learner is estimated and combined with the previous base learners. Thus, the model is equivalent to the traditional gradient descent method, where at each step, a new base learner gives a step in the minimizing direction of a specific risk (loss) function.

More formally, we want to find a function F that minimizes the expected error in predicting the transmittance given the observation of the covariates that are available. We define the following risk function:

(1) $$L\left(F\right)=E_{y,x}\left[(y-F{\left(x\right)}^{2}\right]$$

where the expectation is under the joint distribution of (*y*,*x*) and we are using the squared error as our loss function. In other words, we are seeking the best function that predicts average transmittance given the chemical composition, sintering temperature, density, and thickness of the sample.

Since we have finite data, the risk function defined in Equation (1) must be replaced by the empirical risk function given by Equation (2):

(2) $$R_{F}\left(y,x\right)=\frac{1}{N}\overset{N}{\underset{i=1}{\sum }}L\left(F\left(x_{i}\right),y_{i}\right)$$

where the function *F* is restricted to an additive expansion of the form:

(3) $$F\left(x;{\left\{\rho_{m};\theta_{m}\right\}}_{m=1}^{M}\right)=\overset{M}{\underset{m=1}{\sum }}\rho_{m}h\left(x;\theta_{m}\right).$$

In Equation (3), each function h is the so-called base learner. To explicitly find the function F that minimizes Equation (2), the same methodology proposed by [14] will be used. The descending gradient algorithm is based on the idea of steepest descent, where at each iteration we take a decrease in the direction of decreasing the loss function L.

Assuming that the model was calculated up to the *m−1* iteration, the gradient in the *m-th* iteration is calculated as Equation (4):

(4) $${\hat{u}}_{m}\left(x\right)={\left.\frac{E_{x,y}\left[L(y,F\left(x\right)\right]}{\partial F\left(x\right)}\right|}_{F={\hat{F}}_{m-1}},$$

under mild regularity conditions [11] we have Equation (5):

(5) $${\hat{u}}_{m}\left(x\right)={\left.\frac{\partial R_{F}\left(y,x\right)}{\partial F\left(x\right)}\right|}_{F={\hat{F}}_{m-1}},$$

where in Equation (6):

(6) $${\hat{F}}_{m-1}\left(x;{\left\{{\hat{\rho }}_{k};{\hat{\theta }}_{k}\right\}}_{k=1}^{m-1}\right)=\overset{m-1}{\underset{i=1}{\sum }}{\hat{\rho }}_{i}h_{i}\left(x;{\hat{\theta }}_{i}\right),$$

Once we have the estimation of the gradient $u_{m}\left(x\right)$, a two-step procedure can be followed to estimate the base learner and step size. First we calculate the Equation (7):

(7) $${\hat{\theta }}_{m}=\arg min\overset{N}{\underset{i=1}{\sum }}{\left(-{\hat{u}}_{m}\left(x_{i}\right)-h\left(x_{i};\theta \right)\right)}^{2}.$$

Then, we calculate the total step in the direction of the new base learner $h\left(x;\theta_{m}\right)$ by solving in Equation (8):

(8) $${\hat{\rho }}_{m}=\arg min\overset{N}{\underset{i=1}{\sum }}L\left(y_{i},{\hat{F}}_{m-1}\left(x_{i};{\left\{{\hat{\rho }}_{k};{\hat{\theta }}_{k}\right\}}_{k=1}^{m-1}\right)+\rho h\left(x_{i};{\hat{\theta }}_{m}\right)\right).$$

Finally, the model and forecast in the *m-th* step are given by Equation (9):

(9) $${\hat{F}}_{m}\left(x\right)={\hat{F}}_{m-1}\left(x;{\left\{{\hat{\rho }}_{k};{\hat{\theta }}_{k}\right\}}_{k=1}^{k-1}\right)+{\hat{\rho }}_{m}h\left(x,{\hat{\theta }}_{m}\right)$$

After *M* iterations the model will be given by Equation (10):

(10) $${\hat{F}}_{M}\left(x;{\left\{{\hat{\rho }}_{m};{\hat{\theta }}_{m}\right\}}_{m=1}^{M}\right)=f_{0}+\overset{M}{\underset{m=1}{\sum }}\nu {\hat{\rho }}_{m}h\left(x,{\hat{\theta }}_{m}\right)$$

where *M* is the total base of learners and $f_{0}$ is an initial estimate, which can be zero or the unconditional mean of the data available. We highlight that the parameter ν, called the learning rate, was added for regularization purposes.

For the quantitative analysis of the transmittance results, the modeled variable Y was chosen as the percentage transmittance of each of the zirconia samples tested. The set of analyzed variables were the thickness of the sample, the composition of the zirconia (% yttrium and alumina in mols), the sample density, and the sintering temperature.

In this work, the BooST algorithm was used to estimate the model [16]. The main difference between BooST and other gradient-boosting methods is the use of smooth transition trees (STR-trees) as base learner. STR-trees has a similar form as the conventional classification and regression tree (CART) models with the advantage of being smooth in the decision variable, which allows us to take derivatives. This property will be crucial for the interpretation of the model.

Each STR-tree (base learner) has 4 terminal nodes (parameter). The number of trees used in the model was selected using 5-fold cross validation. To estimate the model, the software R and the algorithm proposed in [16] was used. In the same reference, it is also possible to find the code and link for a GitHub repository where the package is available for free installation.

## 4. Results and Discussion

**Figure 1:**
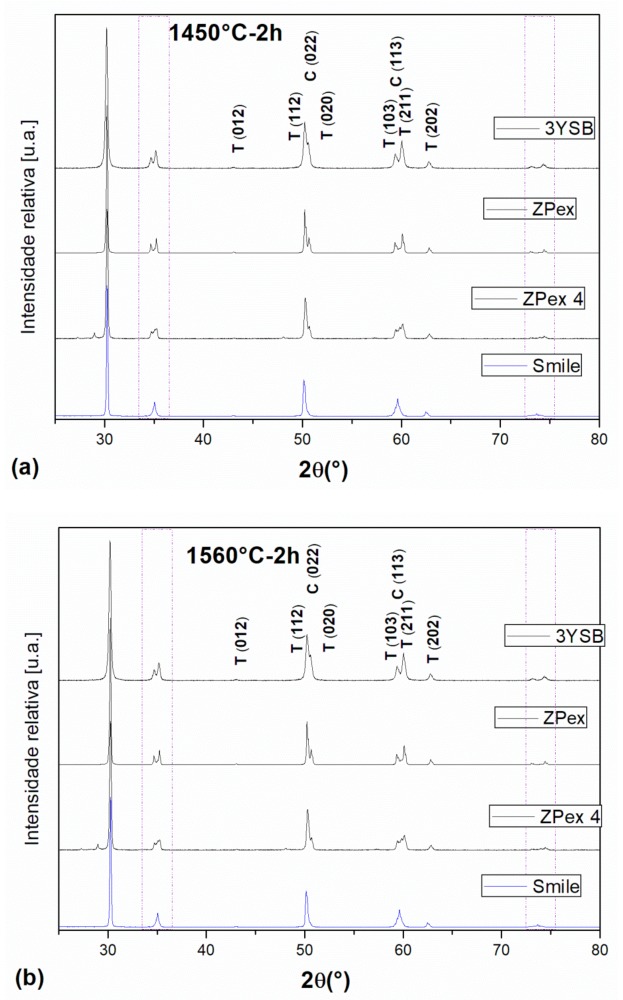

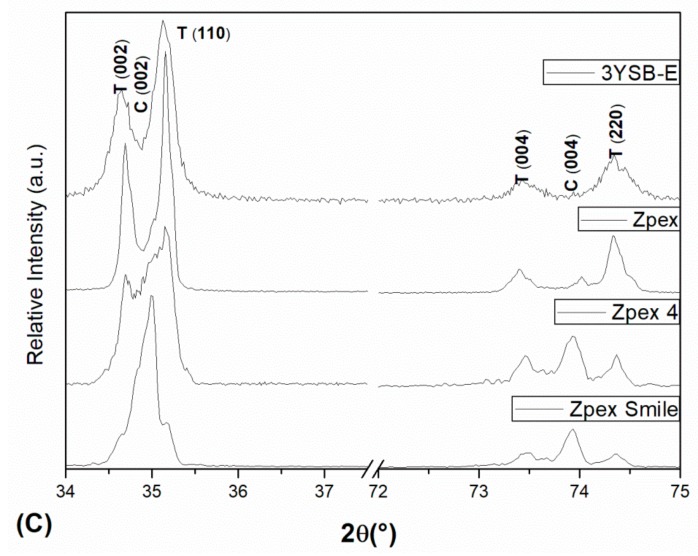
X-ray diffraction patterns (XRD) of different zirconia ceramics for extreme sintering temperatures: (**a**) 1450 °C, (**b**) 1560 °C, and (**c**) detail of 2 theta between 34–37° and 45–70°, the region with the greatest variation of ZrO_2_-cubic and ZrO_2_-tetragonal perceptual.

**Figure 2:**
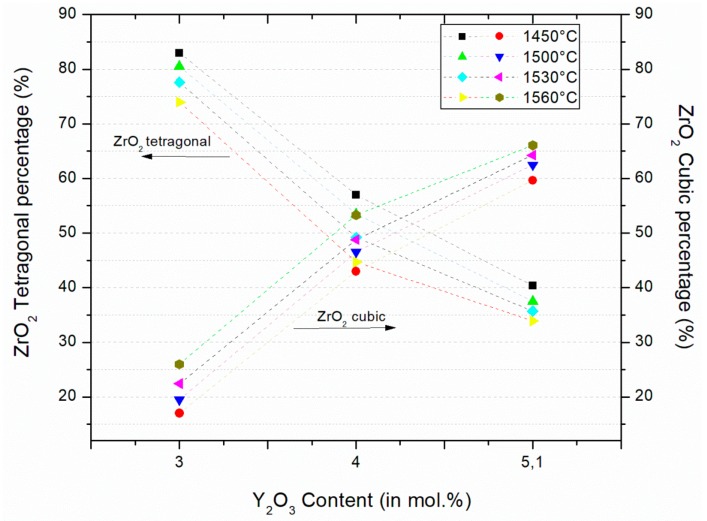
Zirconia tetragonal (T) and cubic (C) phase after sintering at 1450 °C, 1500 °C, 1530 °C, and 1560 °C. Calculation based on [10,11,12].

The results from XRD analysis identified two distinct phases present in all samples (Figure 1). The phase with the highest percentage was tetragonal zirconia (Figure 2). The tetragonal phase was present at levels of about 75–85% of the total crystalline phases, in 3Y-TZP. The second phase was identified as cubic zirconia, which was present in quantities ranging from 15–25%. The percentage of ZrO_2_ cubic phase increases with increasing percentage of Y_2_O_3_.

When comparing the tetragonal phase percentage of sintered 3Y-SBEat different temperatures, it can be observed that the sintered samples at 1450 °C showed the highest percentage of tetragonal phase. The samples sintered at 1560 °C showed the lowest percentage of tetragonal phase. The gap between the highest and the lowest percentage was 9%. For the other groups of zirconia, the differences between the gaps of tetragonal phase after sintering were from 6–10%, as a function of sintering temperature. This result indicates that only the sintering temperature is not statistically significant to modify the relationship between the percentages of tetragonal and cubic phases.

On the other hand, comparing the influence of the variation in the Y_2_O_3_ content (from 3–5.2 mol %) in the composition of the different zirconia, the variations of the percentage of phases were between 40% and 43% among the sintered samples at the same temperature. This result indicates that the influence of yttria content in the percentages of the phases is significant.

**Figure 3:**
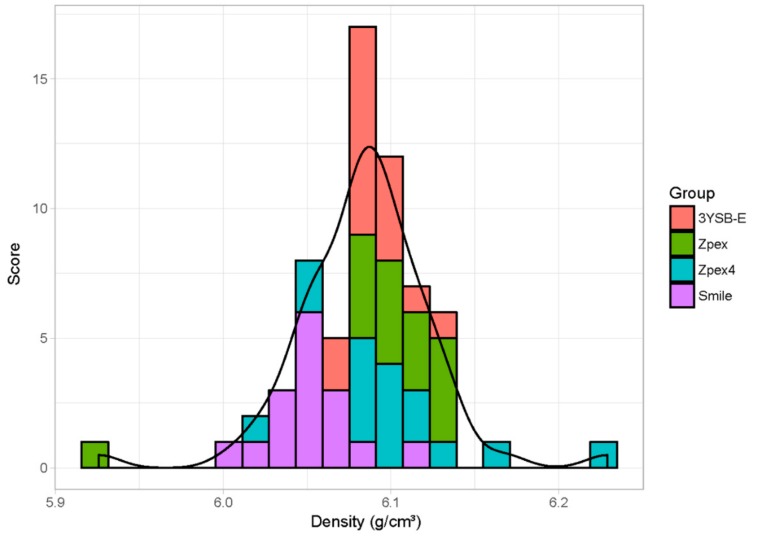
Range of densities for different specimens used in the transmittance test. The colors represent the different types of zirconia.

Before presenting the results obtained with the statistical model, one can see in Figure 3 the dispersion of the apparent density obtained for each type of zirconia after sintering. The dispersion occurs both within groups and within sintering temperatures, which allows separating the effects of density and sintering temperature in the transmittance of the material.

Table 2 shows the roughness parameter of the 3Y-SBE zirconia group samples. The roughnesses of the other groups were similar, and there was no statistically significant difference among groups.

**Figure 4:**
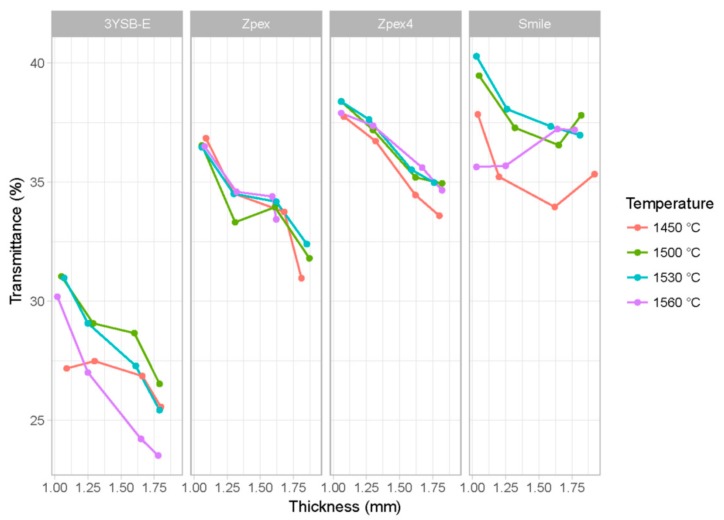
Observed transmittance data for *λ* equal to 710 nm as a function of sintering temperature, zirconia type, and thickness sample.

In Figure 4, the transmittance results are presented as a function of sintering temperature, zirconia type, and thickness. On the *x*-axis, one can see the thickness of the sample. Each facet corresponds to a specific type of zirconia (3Y-SBE, Zpex, Zpex 4, and Zpex-Smile), each color represents a different sintering temperature, and the *x*-axis is the mean thickness of the specimen. The *y*-axis is the value of the transmittance. The wavelength was fixed at 710 nm since changing the wavelength does not affect the relationship of transmittance with the explanatory variables.

Naturally, the thickness variable influences the translucency of the material, since the larger the thickness, the greater the interaction with the factors responsible for scattering the incident electromagnetic wave. It is also expected that the sintering temperature influences the transmittance of the zirconia because it directly affects the grain size and the level of porosity of the sintered zirconia.

Yu et al. [17] determined the translucency parameter (TP) of human and bovine enamel using two spectrophotometers with different aperture sizes. Mean TP values of 1 mm-thick bovine enamel, bovine dentin, human enamel, and human dentin were 14.7, 15.2, 18.7, and 16.4, respectively, based on a 3 mm round aperture. There were significant correlations between the TP values measured by the two apertures; the bigger the aperture size, the higher the TP value (r = 0.87–0.91, *p* < 0.01). The translucency of enamel and dentin increased in direct proportion to wavelength and in inverse proportion to thickness (r = 0.87–0.91).

**Figure 5:**
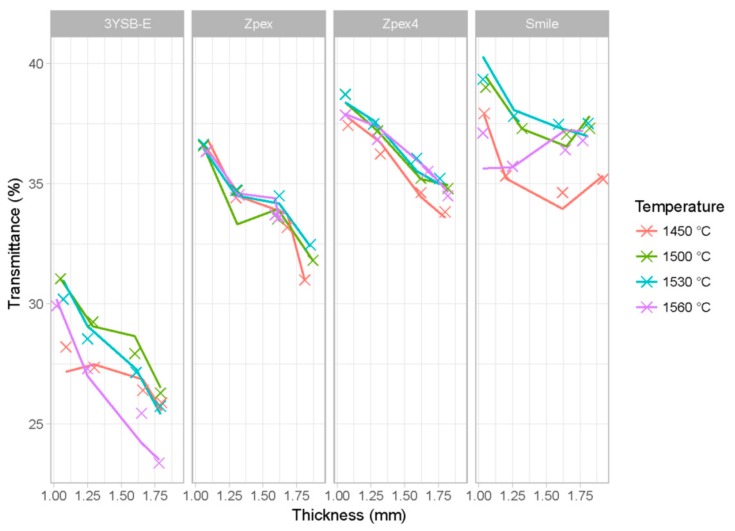
Results adjusted by the model (lines) and observations determined in the experiments (asterisks).

In addition, the final density is also correlated with the level of porosity, and the difference in chemical composition between different types of zirconia affects the type of metastable phase at room temperature, which also affects the transmittance of the specimens. Figure 5 shows the predicted transmittance for the sample data. The asterisks are the experimental values, and lines are the predictions of the model. Each grid corresponds to different types of zirconia. The colors represent different sintering temperatures and the *x*-axis is the thickness of each specimen. The *y*-axis is the value of the transmittance.

In Figure 5, one can see that the predictions of the models are close to the values quantified in the transmittance test, replicating even the convexity observed for the samples of the Zpex-Smile group, caused by the differences in the final density that is not captured by the plot.

**Figure 6:**
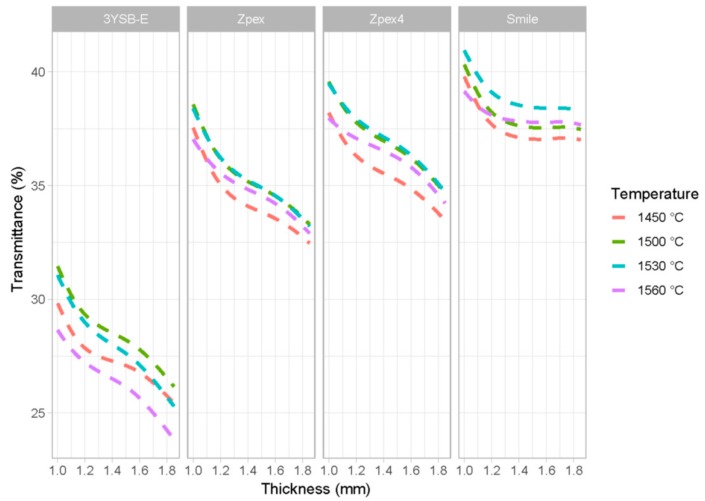
Estimation of transmittance values considering the mean apparent density of the samples.

With the adjusted model, it was possible to analyze the influence of the sintering temperature, the thickness, density, and composition in the transmittance. By fixing a specific density value—say, the average—the other parameters can be varied to understand the impact of these variables on the transmittance. Figure 6 shows the predicted transmittance values considering the mean density of the all specimens.

In Figure 6 it is possible to observe that for the 3Y-SBE, Zpex, and Zpex-4 groups, the influence of the thickness on the transmittance of the sample is approximately linear. For the Zpex-Smile group of zirconia, the linear effect is not observed and the convexity observed for Zpex-Smile was indeed a consequence of different densities for the predicted value for the samples. The main reason for this is that the cubic phase, totally stabilized at room temperature, is isotropic in relation to the transmittance of the electromagnetic wave, significantly reducing the scattering observed in the grain boundary of the monoclinic and tetragonal phases [18].

**Figure 7:**
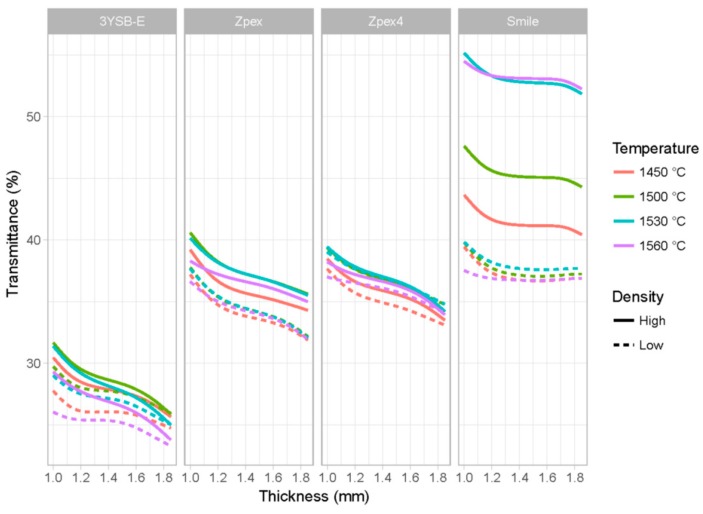
Influence of density and sintering temperature on zirconia transmittance groups.

Figure 7 shows the predicted values of the transmittance for different types of densities—low density (dotted lines) or high density (solid lines). To carry out this analysis, the value of the density was fixed at 2.5 standard deviations either below or above the mean density and then the sensitivity analysis of the other parameters (thickness, sintering temperature, and chemical composition) can proceed.

The model was able to identify the influence of the density for each zirconia group. The difference among groups suggests that the zirconia microstructure plays an important role in how significant the impact of density (and consequently porosity) is on the scattering of the incident light.

For the Zpex-Smile group, the higher sintering temperature and high density generates a zirconia with higher transmittance. For the other types of zirconia, the density also affects the transmittance. However, the partial effect is clearly lower. In addition, it is possible to verify that for the Zpex-Smile zirconia, there is less influence of the sample thickness regardless of density level. However, for the other types of zirconia, the influence of sintering temperature is still approximately linear.

**Figure 8:**
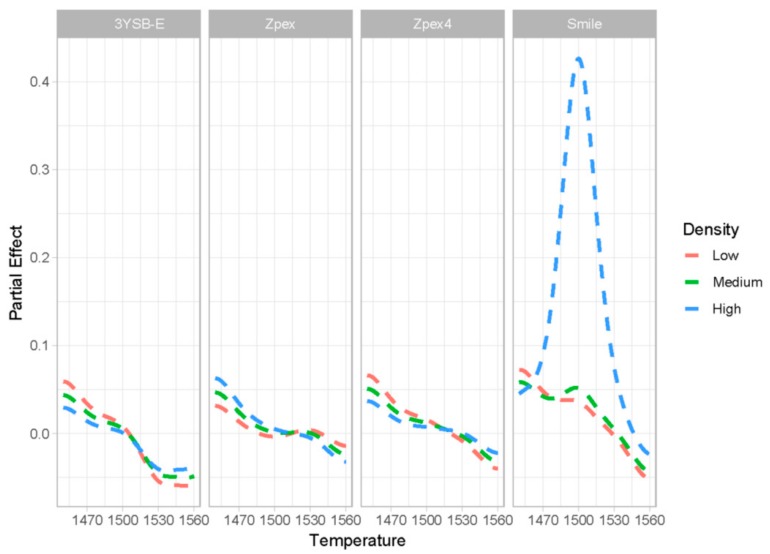
Partial effect of the sintering temperature for each of the zirconia types and with different values of apparent density.

Figure 8 shows the partial influence of the sintering temperature variation on zirconia transmittance. For each group, results were estimated for low-, medium-, and high-density samples. The objective of analyzing the three densities was to verify quantitatively how the density affects the partial effect of the sintering temperature in transmittance. It can be observed that for all zirconia groups, the partial effect starts positive, i.e., as the sintering temperature increases, the transmittance increases (the partial effect is above zero). This result corroborates other works in the literature [18,19,20]. After a certain temperature, which varies with the type of zirconia, increasing the sintering temperature leads to a decrease in transmittance (partial effects are negative). The hypothesis for the decrease of the transmittance as the sintering temperatures increase can be associated with the nucleation of microcracks in grain boundary at high temperatures due the grain growing. This has also been described in the literature [19,20,21].

Based on the results obtained by the proposed methodology, it is observed that at lower sintering temperatures than that for maximum densification, the positive effect of grain size decreases. Above a certain sintering temperature, grain growth induces microcrack nucleation and decreases the novel zirconia for dentistry (NZD) transmittance with a negative effect.

The results of the mathematical models showed a higher transmittance sensitivity of the sintered NZD-4 at higher temperature. For this group, if the zirconia reached the expected maximum densification around 1550 °C, where the highest transmittance is obtained. If the Zpex-Smile has not yet reached the maximum densification, the sensitivity in relation to the sintering temperature is similar to that for the other groups.

It also worth noting that for the zirconia Zpex-Smile, the partial effect of the sintering temperature is surprisingly high. Specifically, for this type of zirconia, the model points out that high density and high sintering temperature are both fundamental for the best transmittance results of the material.

Based on the data of the roughness parameters shown in Table 2 and considering that there was no difference in the roughness among the groups, it is possible to consider that the surface finish of the samples did not influence the transmittance. Before sintering, all samples were polished under the same conditions.

Even though the grain size is known to be an important parameter in the transmittance of zirconia, the dependence of the grain size with the sintering temperature and the powder microstructure is well established [22] and sufficiently smooth. Therefore, since we are already controlling for the sintering temperature and type of zirconia are already controlled, there is no need, statistically speaking, to also include the grain size in the statistical model that is described in the sequence.

One analysis of interest is to understand the partial effect of the grain size in the transmittance of zirconia. However, for practical use, the control over the sintering temperature and type of zirconia is easier, especially for practical applications, since it avoids the use of techniques such as SEM and indirectly measuring the same effect.

## 5. Summary and Conclusions

Based on experimental results and in the statistical learning method proposed, it was possible to analyze the effect of the sintering temperature in different types of zirconia commercially available. The proposed statistical model was fundamental to identify the nonlinear dependencies among the sintering temperature, chemical composition, density, and thickness of the samples in the zirconia transmittance.

The sensibility analysis presented no linear dependence between transmittance and specimen thickness for 3YSBE, Zpex, and Zpex-4, and a sublinear dependence for zirconia Zpex-Smile. Increasing the sintering temperature has a positive impact in the transmittance of the zirconia up to a certain level, which varies with density and chemical composition. After this point, increasing the sintering temperature decreases the transmittance of the material. The group deriving the greatest benefit from high sintering temperature was zirconia Zpex-Smile.

## Figures and Tables

**Table 1:** Powder chemical composition (in wt %) used to produce the zirconia blocks (according manufacturing data).

Zirconia Powder	Designation	Zr_2_O_3_	Al_2_O_3_	Y_2_O_3_	SiO_2_	Fe_2_O_3_
TZ-3YSBE	NZD-1	94.72	0.055	5.2	<0.02	<0.01
Zpex	NZD-2	94.72	0.055	5.2	<0.02	<0.01
Zpex-4	NZD-3	92.96	0.050	7.0	<0.02	<0.01
Zpex-Smile	NZD-4	90.62	0.059	9.3	<0.02	<0.01

**Table 2:** Roughness of 3Y-SBE group after sintered at different temperatures (°C).

Sintering Temperature (°C)	Statistical	Ra (µm)	PV (µm)	Peak (µm)	Valley (µm)
1450	Mean	0.160	7.566	5.92	−1.65
	Std Dev	0.011	2.906	2.80	0.52
1500	Mean	0.170	4.702	2.76	−1.95
	Std Dev	0.010	1.483	1.74	0.31
1530	Mean	0.146	3.111	1.88	−1.23
	Std Dev	0.012	0.639	0.69	0.08
1560	Mean	0.203	11.241	8.28	−2.96
	Std Dev	0.016	1.889	1.98	0.60
